# Association between serum vitamin D and the risk of diabetic kidney disease in patients with type 2 diabetes

**DOI:** 10.3389/fmed.2024.1445487

**Published:** 2024-08-09

**Authors:** Yujie Wang, Chenggang Hu, Ying Li, Qi Liu, Lichao Gao, Dongmei Zhang, Ling Cao

**Affiliations:** ^1^Department of Nephrology, Affiliated Hospital of Southwest Medical University, Luzhou, Sichuan, China; ^2^Sichuan Clinical Research Center for Nephropathy, Luzhou, Sichuan, China; ^3^Emergency Department, The Affiliated TCM Hospital of Southwest Medical University, Luzhou, Sichuan, China

**Keywords:** diabetic kidney disease, type 2 diabetes mellitus, vitamin D, mortality, non-linear relationship

## Abstract

**Aims:**

This investigation explored the potential correlation between serum vitamin D concentration and diabetic kidney disease (DKD) among patients with type 2 diabetes mellitus (T2DM).

**Methods:**

This cross-sectional study assessed 4,570 patients with T2DM drawn from the National Health and Nutrition Examination Survey (NHANES) dataset. Restricted cubic splines were utilized to examine the dose–response relationship between serum vitamin D levels and the risk of DKD in patients with T2DM. Serum vitamin D concentrations were divided into quartiles for multivariable logistic regression analysis to evaluate the association between varying serum vitamin D levels and DKD risk in patients with T2DM. Additionally, sex-stratified analyses were conducted to determine consistency of the results. The influence of vitamin D concentrations on mortality risk was assessed using a Cox regression model.

**Results:**

Of the patients with T2DM, 33% were diagnosed with DKD. Restricted cubic spline plots revealed a U-shaped relationship between vitamin D levels and DKD risk, with a protective effect noted in the mid-range, indicating optimal serum vitamin D concentrations between 59.6 nmol/L and 84.3 nmol/L. The multivariate Cox regression analysis suggested that higher VID levels were associated with a reduced mortality risk, particularly in male patients.

**Conclusion:**

The regulation and monitoring of serum vitamin D levels within an optimal range may play a pivotal role in the prevention of DKD in patients with T2DM. Public health strategies should emphasize the regular monitoring of vitamin D levels, especially among populations at elevated risk, to mitigate the progression of DKD and decrease the associated mortality rates.

## 1 Introduction

In recent years, type 2 diabetes mellitus (T2DM) has escalated considerably owing to factors such as improved economic conditions and lifestyle changes. By 2045, the global diabetic population is estimated to reach 700 million (1). T2DM disrupts carbohydrate and lipid metabolism and damages several organs, including the kidneys. Diabetic kidney disease (DKD), a prevalent chronic microvascular complication of diabetes, impacts approximately one-third of all individuals with T2DM (2, 3). DKD is the primary cause of end-stage kidney disease, representing >50% of such cases (4). Often, those with chronic kidney disease (CKD) or end-stage kidney disease (ESKD) require kidney transplantation or dialysis, which severely impacts quality of life and increases the risk of cardiovascular diseases and mortality (5).

Vitamin D, a fat-soluble micronutrient that the skin synthesizes through ultraviolet B radiation, is essential for human health. It places a crucial role in promoting insulin synthesis and secretion and enhancing insulin sensitivity, thereby aiding in maintaining metabolic balance (6, 7). Lowered vitamin D levels are closely associated with elevated blood glucose levels, insulin resistance, and microvascular complications (8, 9). Furthermore, significant evidence linking serum vitamin D levels with the development of DKD is available. Epidemiological evidence indicates that vitamin D deficiency is associated with higher mortality rates in patients with T2DM and kidney disease, regardless of other confounding variables (10, 11). In animal models, 1,25-dihydroxyvitamin D3 and its analogs have been shown to reduce renal inflammation and fibrosis, inhibit the renin-angiotensin system, support podocyte survival, and decrease albuminuria and glomerulosclerosis (12–14). Additionally, vitamin D ameliorates podocyte injury by enhancing autophagy activity in diabetic nephropathy (15).

Despite the critical role of vitamin D in managing diabetes and DKD, deficiencies remain widespread owing to changes in lifestyle and dietary structures (16). Approximately one billion people globally suffer from vitamin D deficiency, with up to 57% of American adults affected (17). Investigating the potential role of vitamin D in preventing the development and progression of DKD in patients with T2DM is crucial for its early prediction and prevention. Identifying the risk factors that influence DKD severity is essential for developing medical strategies to mitigate disease progression and prevent renal failure. However, the association between serum vitamin D levels and DKD in patients with T2DM has not yet been studied in a nationally representative sample of American adults. Therefore, we conducted a retrospective analysis using data from the National Health and Nutrition Examination Survey (NHANES) database from 2009 to 2018 to explore the impact of serum vitamin D levels on the development of DKD.

## 2 Methods

### 2.1 Study population

This study used data from the NHANES dataset, which aims to assess the demographic, health, and nutritional information in the United States and is designed to be nationally representative of the noninstitutionalized civilian population in the United States.

The survey includes demographic, dietary, examination, and laboratory data collected by trained personnel. The survey received ethical clearance from the relevant ethics committee, and informed consent was given by all participants. Detailed documentation of NHANES methodology and data is publicly available at the Centers for Disease Control and Prevention (CDC) website.^1^

**Figure 1:**
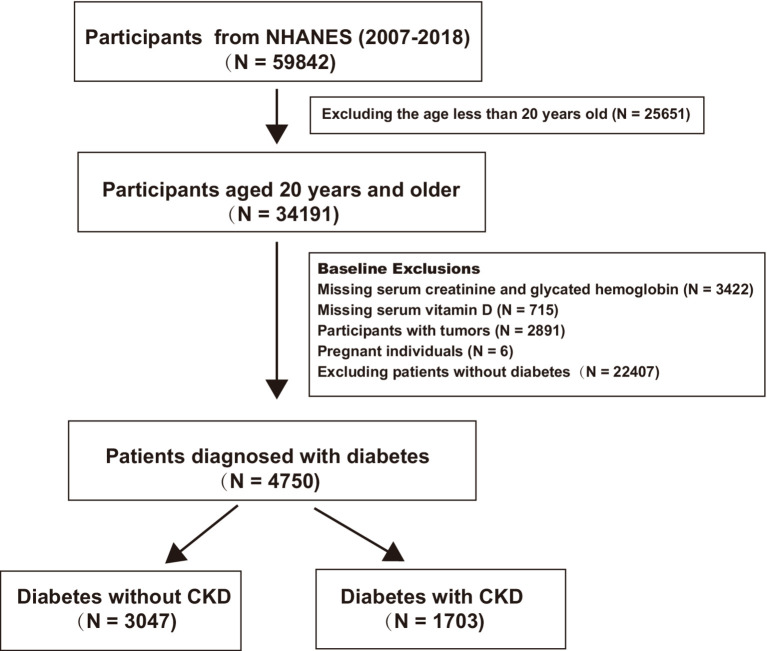
Flow chart of queue participant selection.

For this analysis, data from six continuous NHANES cycles spanning from 2007 to 2018 were included, involving 59,842 participants. The inclusion criteria were adults aged ≥20 years diagnosed with T2DM, defined either by a physician’s diagnosis or by laboratory results showing a fasting plasma glucose level of ≥7.0 mmol/L or glycated hemoglobin value of ≥6.5%. The exclusion criteria were individuals with cancer, pregnant women, and those missing hemoglobin, creatinine, or vitamin D data. DKD was defined as occurring in patients with diabetes with estimated glomerular filtration rates (eGFRs) of <60 mL/min/1.73 m^2^ or the presence of albuminuria (albumin-to-creatinine ratio of ≥30 mg/g). The creatinine equation from the Chronic Kidney Disease Epidemiology Collaboration (CKD-EPI) was utilized to estimate the eGFR, factoring in age, sex, race, and serum creatinine levels (18). After screening, 4,750 patients with diabetes were included. Of these, 1,073 had concurrent DKD, while 3,047 did not. Figure 1 provides a summary of the inclusion and exclusion of the participants.

### 2.2 Study variables

Serum vitamin D levels were measured using ultra-high performance liquid chromatography–tandem mass spectrometry at the NHANES laboratory. Serum vitamin D concentrations were categorized into quartiles. Vitamin D status was also divided into four groups according to the Endocrine Society’s clinical practice guidelines: sufficiency (≥75.0 nmol/L), insufficiency (50.0–74.9 nmol/L), moderate deficiency (25.0–49.9 nmol/L), and severe deficiency (<25.0 nmol/L) (19).

Covariates considered in the analysis included demographics (age, sex, race), socioeconomic factors (education level, marital status, family income), body mass index (BMI), lifestyle factors (alcohol consumption, smoking status), medical history (hypertension, medication use), and laboratory results (blood sugar, eGFR, albumin-to-creatinine ratio). Racial/ethnic categories included non-Hispanic Black, Mexican American, non-Hispanic White, other Hispanic, and other multiracial. Education levels were categorized as high school or less, some college, and college graduate or above. Family income was expressed as a ratio to the poverty threshold (0–1.3, 1.3–3.5, >3.5) (20). Alcohol consumption was categorized as non-drinkers, moderate drinkers (1–5 drinks per month), advanced drinkers (5–10 drinks per month), and heavy drinkers (>10 drinks per month). Smoking status was classified into never smokers, former smokers, and current smokers. Hypertension was identified by a systolic blood pressure of ≥140 mmHg, a diastolic blood pressure of ≥90 mmHg, or a previous diagnosis.

### 2.3 Statistical analyses

Given the inherent complex design of the NHANES survey, all analyses were conducted using a weighted approach as recommended by NHANES guidelines. Patient baseline characteristics for T2DM with and without DKD were analyzed using weighted chi-square tests for categorical data and weighted Student’s *t*-tests for continuous data. The relationship between serum vitamin D levels and the presence of diabetic kidney disease (DKD) was examined through both univariate and multivariate weighted logistic regression analyses. This observational study was structured around three statistical models in alignment with the STROBE guidelines. Model 1 was unadjusted; Model 2 was adjusted for sex, age, and race/ethnicity; Model 3 included the Model 2 adjustments plus additional adjustments for BMI, education level, smoking status, alcohol consumption, income-to-poverty ratio, use of diabetes medication, hypertension, glycated hemoglobin (HbA1c) and types of hypoglycemic drugs.

Univariate analyses provided initial insights into the direct association without adjusting for confounders, while multivariate logistic analyses accounted for a comprehensive set of covariates, including demographic factors, socioeconomic status, lifestyle behaviors, medical history, and laboratory results, to assess the independent effect of serum vitamin D levels on DKD risk. Weighted Cox proportional hazards models were employed to assess the hazard ratios and 95% confidence intervals (CIs) for the association between serum vitamin D levels and overall survival across these different models.

Subgroup analyses based on sex were conducted to evaluate the heterogeneity of associations across different groups, aiming to understand if the relationships between serum vitamin D levels and DKD risk vary significantly between male and female patients. Furthermore, to investigate the dose–response correlation between serum vitamin D levels and the risk of DKD in patients with type 2 diabetes mellitus (T2DM), a restricted cubic spline (RCS) regression was utilized. This method involved positioning knots at the 5th, 50th, and 75th percentiles of serum vitamin D levels to accurately capture potential non-linear relationships. Adjustments in this analysis were comprehensive, incorporating all covariates from Model 3, including demographic variables, socioeconomic factors, lifestyle factors, medical history, and laboratory results, to ensure robustness and minimize confounding effects.

Model validation included the assessment of multicollinearity using the Variance Inflation Factor (VIF), ensuring that no variable had a VIF greater than 10. For the multivariable logistic regression model (Model 3), we plotted the Receiver Operating Characteristic (ROC) curve and calculated the Area Under the Curve (AUC) to evaluate the model’s discriminatory power. Sensitivity analysis was conducted by re-estimating the model after excluding patients older than 80 years to test the robustness of the results.

Statistical analyses were performed using R software, version 3.6.3, developed by the R Core Team in Vienna, Austria. Statistical significance was set at a *p*-value of <0.05.

## 3 Results

### 3.1 Demographic characteristics

This study included 4,750 patients with diabetes, with a median age of 58 years. The cohort comprised 2,253 female (47%) and 2,497 male (53%) patients. The weighted baseline characteristics of study participants is shown in Table 1. The median age in the DKD group was notably greater than that in the non-DKD group (65 years versus 56 years; *p* < 0.001).

**Table 1:** Demographic characteristics of patients.

Characteristic	Overall 4,750 (100%)	T2D patients without DKD 3,047 (67%)	T2D patients with DKD 1703 (33%)	*p* value
**Age**	58 (49, 68)	56 (47, 64)	65 (53, 74)	**<0.001**
**BMI**	32 (28, 37)	32 (28, 37)	32 (28, 38)	0.11
**Sex**				0.60
*Female*	2,253 (47%)	1,467 (46%)	786 (48%)	
*Male*	2,497 (53%)	1,580 (54%)	917 (52%)	
**Age.group**				**<0.001**
*<60 years*	2,061 (54%)	1,571 (62%)	490 (37%)	
*60+ years*	2,689 (46%)	1,476 (38%)	1,213 (63%)	
**Race**				0.50
*Mexican American*	918 (11%)	619 (11%)	299 (11%)	
*Other Hispanic*	569 (6.6%)	401 (7.0%)	168 (5.8%)	
*Non-Hispanic White*	1,424 (56%)	849 (56%)	575 (57%)	
*Non-Hispanic Black*	1,237 (16%)	785 (15%)	452 (16%)	
*Other/multiracial*	602 (10%)	393 (10%)	209 (10%)	
**Education**				**0.015**
*High school or less*	2,779 (50%)	1,742 (47%)	1,037 (54%)	
*Some College*	1,242 (30%)	800 (31%)	442 (29%)	
*College Graduate or above*	729 (20%)	505 (22%)	224 (16%)	
**family.income**				**<0.001**
*High income*	1,083 (34%)	772 (38%)	311 (27%)	
*Low income*	1,852 (28%)	1,135 (26%)	717 (33%)	
*Medium income*	1,815 (37%)	1,140 (36%)	675 (40%)	
**Marital_status**				**<0.001**
*Married*	2,584 (58%)	1,723 (60%)	861 (53%)	
*Never married*	510 (11%)	346 (12%)	164 (9.0%)	
*Living with partner*	230 (5.5%)	165 (5.7%)	65 (5.3%)	
*Other*	1,426 (26%)	813 (22%)	613 (33%)	
**Alq.group**				**0.018**
*Non-drinker*	1,708 (32%)	1,055 (30%)	653 (35%)	
*1–5 drinks/month*	2,419 (54%)	1,568 (54%)	851 (53%)	
*5–10 drinks/month*	216 (4.7%)	149 (5.2%)	67 (3.7%)	
*10+ drinks/month*	407 (9.8%)	275 (11%)	132 (8.4%)	
**Smoke.group**				**<0.001**
*Never smoker*	2,440 (50%)	1,599 (51%)	841 (48%)	
*Former smoker*	1,503 (33%)	888 (30%)	615 (38%)	
*Current smoker*	807 (17%)	560 (19%)	247 (14%)	
**BMI.group**				0.066
*Normal (18.5 to < 25)*	628 (11%)	381 (11%)	247 (12%)	
*Obese (30 or greater)*	2,769 (63%)	1,757 (62%)	1,012 (65%)	
*Overweight (25 to < 30)*	1,337 (25%)	899 (27%)	438 (22%)	
*Underweight (<18.5)*	16 (0.2%)	10 (0.2%)	6 (0.3%)	
**HBP**				**<0.001**
*No hypertension*	983 (22%)	771 (25%)	212 (15%)	
*Hypertension*	3,767 (78%)	2,276 (75%)	1,491 (85%)	
**Serum vitamin D levels**	63 (46, 83)	63 (46, 80)	64 (44, 86)	0.30
**Serum vitamin D quartile group**				**0.001**
*Q1*	1,398 (25%)	901 (24%)	497 (27%)	
*Q2*	1,240 (25%)	827 (26%)	413 (23%)	
*Q3*	1,076 (25%)	745 (27%)	331 (21%)	
*Q4*	1,036 (25%)	574 (23%)	462 (29%)	
**eGFR**	89 (69, 103)	94 (82, 105)	61 (49, 93)	**<0.001**
**URDACT**	11 (6, 29)	8 (5, 13)	52 (27, 161)	**<0.001**
**Treat**				**<0.001**
*Only diabetes medicines*	1,752 (38%)	1,129 (37%)	623 (39%)	
*Any insulin*	996 (21%)	464 (17%)	532 (30%)	
*No drug*	736 (17%)	504 (18%)	232 (15%)	
*other*	1,266 (24%)	950 (28%)	316 (16%)	

N not Missing (unweighted); Median (IQR) for continuous; *n* (%) for categorical; Wilcoxon rank-sum test for complex survey samples; chi-squared test with Rao & Scott’s second-order correction. *p*-values in bold represent significant variables with values less than 0.05.

The average estimated glomerular filtration rates (eGFR) were 94 for the DKD group and 61 for the non-DKD group. Levels of education and family income were greater in the non-DKD group than in the DKD group. Furthermore, 52% of patients with DKD had histories of smoking or were current smokers, while moderate drinking was more common among patients without DKD. The median and interquartile range of the serum vitamin D level was 63 (46, 83) nmol/L; the level did not differ between the non-DKD and DKD groups (63 [46, 80] nmol/L vs. 64 [44, 86] nmol/L, *p* = 0.30). In the study, 32% of patients in the DKD group showed a severe deficiency in vitamin D (levels below 50 nmol/L), as opposed to 29.7% in the non-DKD group.

### 3.2 Factors influencing DKD incidence

Overall, 33% of the patients were diagnosed with DKD. The serum vitamin D levels were quartiled to analyze the independent effects of vitamin D levels on DKD; the corresponding vitamin D values of the 25, 50, and 75% quartiles were 45.6, 63.2, and 82.7 nmol/L, respectively. Multiple models were created to assess this relationship.

**Figure 2:**
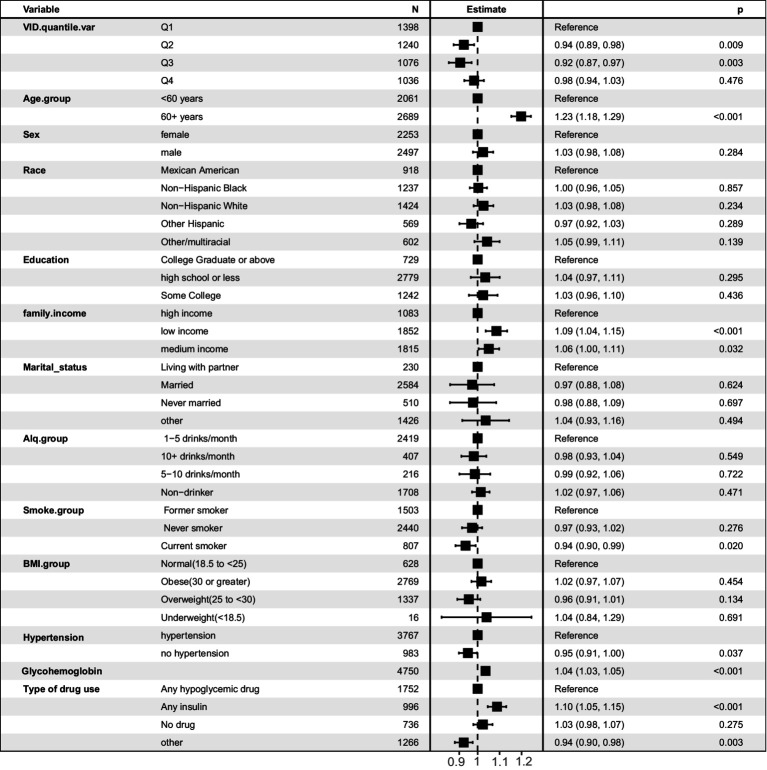
Forest plot of multivariate logistic regression analysis predicting diabetic nephropathy risk based on multiple variables.

In the univariate logistic regression analysis, higher serum vitamin D levels were a protective factor against DKD (Table 2). Compared with that of the reference group (first quartile), the odds ratios (ORs) for developing DKD were 0.95 (95% CI, 0.91–1.00) for the second quartile, 0.93 (95% CI, 0.88–0.99) for the third quartile, and 1.03 (95% CI, 0.98–1.08) for the fourth quartile. In Model 2, as shown in Supplementary Table S1, the VIFs for all variables were less than 10. After adjusting for age, sex, and race, the ORs were 0.93 (95% CI, 0.89–0.98), 0.91 (95% CI, 0.85–0.96), and 0.96 (95% CI, 0.91–1.01) for the second, third, and fourth quartiles, respectively. When including all potential confounding factors, race variable has a high Variance Inflation Factor (VIF) as showed in Supplementary Table S2, so we excluded race from other variables in the final model 3, and all variables had expansion factors less than 10 without potential collinearity effects as shown in Supplementary Table S3. In the final model 3, the ORs were 0.94 (95% CI, 0.89–0.98), 0.92 (95% CI, 0.87–0.97), and 0.98 (95% CI, 0.94–1.03) for the second, third, and fourth quartiles, respectively. The multivariate results are shown in Figure 2. Older age is a risk factor for diabetic nephropathy with an OR of 1.23 (95% CI, 1.18, 1.29). Low family income is also a risk factor for diabetic nephropathy with an OR of 1.09 (95% CI, 1.04, 1.15). Additionally, glycated hemoglobin is a risk factor for diabetic nephropathy with an OR of 1.04 (95% CI, 1.03, 1.05).

**Table 2:** Risk factors for DKD in patients with DM.

	Model 1			Model 2			Model 3		
Characteristic	OR	95% CI	*p*-value	OR	95% CI	*p*-value	OR	95% CI	*p*-value
All									
Q1	–	–							
Q2	0.95	0.91, 1.0	0.029	0.93	0.89, 0.98	0.007	0.94	0.89, 0.98	0.009
Q3	0.93	0.88, 0.99	0.014	0.91	0.85, 0.96	<0.001	0.92	0.87, 0.97	0.003
Q4	1.03	0.98, 1.08	0.300	0.96	0.91, 1.01	0.093	0.98	0.94, 1.03	0.476
Male									
Q1	–	–		–	–		–	–	
Q2	0.95	0.90, 1.01	0.087	0.93	0.88, 0.99	0.029	0.93	0.88, 0.99	0.020
Q3	0.91	0.85, 0.97	0.007	0.88	0.82, 0.95	0.001	0.9	0.85, 0.97	0.004
Q4	1.01	0.94, 1.07	0.900	0.95	0.88, 1.03	0.200	0.98	0.92, 1.05	0.648
Female									
Q1	–	–		–	–		–	–	
Q2	0.94	0.87, 1.01	0.100	0.94	0.87, 1.01	0.090	0.95	0.87, 1.03	0.184
Q3	0.96	0.88, 1.05	0.400	0.94	0.86, 1.02	0.140	0.95	0.87, 1.04	0.247
Q4	1.04	0.96, 1.14	0.300	0.96	0.89, 1.05	0.400	0.99	0.91, 1.07	0.741

CI, confidence interval. Model 1 was unadjusted; Model 2 was adjusted for sex, age, and race/ethnicity; Model 3 included the Model 2 adjustments and adjustments for BMI, education level, smoking status, alcohol consumption, income-to-poverty ratio, use of diabetes medication, and hypertension.

Additionally, sensitivity analysis was conducted by excluding individuals older than 80 years. As shown in Supplementary Figure S3, similar conclusions were obtained in the population under 80 years old, with vitamin D being a protective factor. Compared with the reference group (first quartile), the odds ratios (ORs) for developing DKD were 0.94 (95% CI, 0.89–0.99) for the second quartile, 0.93 (95% CI, 0.88–0.98) for the third quartile, and 0.97 (95% CI, 0.92–1.02) for the fourth quartile. Older age, low income, and high glycated hemoglobin levels were identified as risk factors (*p* < 0.05). Additionally, obesity was found to be a risk factor for diabetic kidney disease, with an OR of 1.14 (95% CI, 1.04–1.25). Furthermore, we assessed the model’s accuracy using the area under the ROC curve. As shown in Supplementary Figure S4, the AUC was 0.703 for the entire population and 0.697 for the population under 80 years old.

Stratified by sex, the ORs in male patients were 0.95 (95% CI, 0.90–1.01), 0.91 (95% CI, 0.85–0.97), and 1.01 (95% CI, 0.94–1.07) for the second, third, and fourth quartiles, respectively, compared with that of the first quartile. After adjusting for multiple potential confounding factors, the ORs were 0.94 (95% CI, 0.87–1.01), 0.96 (95% CI, 0.88–1.05), and 1.04 (95% CI, 0.96–1.14). In the male subgroup, the multivariate results shown in Supplementary Figure S2 indicate that older age is a risk factor for diabetic nephropathy with an OR of 1.08 (95% CI, 1.02, 1.13). Low family income is also a risk factor with an OR of 1.23 (95% CI, 1.16, 1.31). Glycated hemoglobin is a risk factor with an OR of 1.04 (95% CI, 1.03, 1.05), and obesity is a risk factor with an OR of 1.04 (95% CI, 1.02, 1.05). However, the protective factors were less pronounced in female patients, with ORs of 0.95 (95% CI, 0.87–1.03), 0.95 (95% CI, 0.87–1.04), and 0.99 (95% CI, 0.91–1.07) for the second, third, and fourth quartiles, respectively.

Analyses based on the severity of vitamin D deficiency were also performed. Compared with that of severe deficiency, the ORs of developing DKD were 0.95 (95% CI, 0.87–1.04) for moderate deficiency, 0.88 (95% CI, 0.81–0.96) for insufficiency, and 0.91 (95% CI, 0.83–1.00) for sufficiency in the overall population. In the male subgroups, compared with that of severe deficiency, the ORs were 0.92 (95% CI, 0.80–1.05) for moderate deficiency, 0.84 (95% CI, 0.74–0.95) for insufficiency, and 0.87 (95% CI, 0.77–0.99) for sufficiency.

### 3.3 Non-linear relationship between serum vitamin D levels and the risk of DKD in patients with T2DM

**Figure 3:**
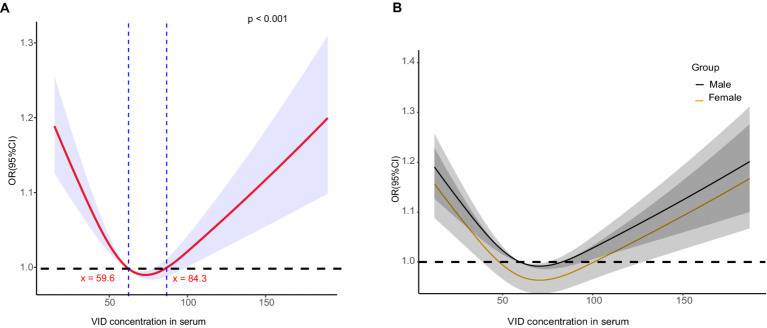
Correlation between serum vitamin D content and diabetic nephropathy.

To determine if a non-linear relationship exists between serum vitamin D levels and diabetic kidney disease (DKD) in patients with type 2 diabetes mellitus (T2DM), restricted cubic spline analysis was used. The analysis aimed to investigate potential non-linear associations between serum vitamin D levels and kidney parameters. A non-linearity of <0.05 indicated a non-linear relationship. The results revealed a U-shaped curve in the risk of DKD relative to the serum vitamin D level (Figure 3). Initially, as the vitamin D level increased, the risk factors for DKD in patients with T2DM decreased. However, at levels >84 nmol/L, an increased vitamin D level was significantly associated with elevated risk factors for DKD in patients with T2DM. Further stratified by gender, the U-shaped curve was more pronounced in female patients, showing that the protection range for female patients (48–100 nmol/L) was wider than for male patients (60–80 nmol/L). DKD risk factors increased significantly when vitamin D levels were > 100 nmol/L in women, and increased significantly when vitamin D levels were > 80 nmol/L in men.

### 3.4 Association between serum vitamin D levels and overall survival

The relationship between serum vitamin D levels and mortality among patients with diabetes was also analyzed (Table 3). In the overall population, before adjusting for confounders, the mortality risks were 0.77 (95% CI, 0.60–1.00) for the second quartile, 0.73 (95% CI, 0.57–0.93) for the third quartile, and 1.28 (95% CI, 0.97–1.68) for the fourth quartile. After adjusting for multiple potential confounders, compared with the reference group (first quartile), the mortality risks were 0.65 (95% CI, 0.51–0.84), 0.54 (95% CI, 0.42–0.69), and 0.80 (95% CI, 0.62–1.02) for the second, third, and fourth quartiles, respectively.

**Table 3:** Risk factors for overall survival in patients with DM.

	Model 1			Model 2			Model 3		
Characteristic	HR	95% CI	*p*-value	HR	95% CI	*p*-value	HR	95% CI	*p*-value
All									
Q1	–	–		–	–		–	–	
Q2	0.77	0.60, 1.00	0.047	0.66	0.52, 0.85	0.001	0.65	0.51, 0.84	0.001
Q3	0.73	0.57, 0.93	0.010	0.53	0.42, 0.68	<0.001	0.54	0.42, 0.69	<0.001
Q4	1.28	0.97, 1.68	0.079	0.71	0.55, 0.93	0.013	0.80	0.62, 1.02	0.076
Male									
Q1	–	–		–	–		–	–	
Q2	0.79	0.57, 1.09	0.200	0.62	0.44, 0.89	0.009	0.62	0.46, 0.85	0.002
Q3	0.74	0.53, 1.04	0.080	0.5	0.35, 0.71	<0.001	0.51	0.36, 0.72	<0.001
Q4	1.18	0.83, 1.68	0.300	0.59	0.41, 0.84	0.004	0.73	0.53, 1.01	0.055
Female									
Q1	–	–		–	–		–	–	
Q2	0.75	0.50, 1.12	0.200	0.67	0.44, 1.01	0.058	0.63	0.41, 0.97	0.038
Q3	0.69	0.44, 1.09	0.110	0.54	0.35, 0.83	0.005	0.54	0.36, 0.82	0.004
Q4	1.37	0.90, 2.10	0.140	0.83	0.54, 1.28	0.400	0.88	0.59, 1.31	0.500

HR, hazard ratio, CI, confidence interval. Model 1 was unadjusted; Model 2 was adjusted for sex, age, and race/ethnicity; Model 3 included the Model 2 adjustments and adjustments for BMI, education level, smoking status, alcohol consumption, income-to-poverty ratio, use of diabetes medication, and hypertension.

In male subgroups, after including multiple potential confounders, the mortality risks compared with those of the reference group were 0.62 (95% CI, 0.46–0.85), 0.51 (95% CI, 0.36–0.72), and 0.73 (95% CI, 0.53–1.01) for the second, third, and fourth quartiles, respectively. In female subgroups, after adjusting for confounders, the mortality risks were 0.63 (95% CI, 0.41–0.97), 0.54 (95% CI, 0.36–0.82), and 0.88 (95% CI, 0.59–1.31) for the second, third, and fourth quartiles, respectively.

Further linear regression analysis revealed a linear correlation between serum vitamin D levels and mortality risk. As serum vitamin D levels increased, the mortality risk decreased. Importantly, serum vitamin D levels exceeding 59.64 nmol/L were associated with a significantly lower mortality risk. When examining the data by sex, it was found that higher serum vitamin D levels in male patients were linked to a reduced mortality risk, especially for levels above 59.64 nmol/L. A similar trend was observed in female patients.

## 4 Discussion

Diabetes mellitus is a metabolic condition that can cause significant damage to various organs, especially the nerves and blood vessels, if not properly managed. The World Health Organization recognizes diabetes as a leading cause of renal failure. In this study, 33% of diabetes patients developed diabetic kidney disease (DKD). Current therapies for DKD focus on managing blood glucose, blood pressure, and lipids, using angiotensin-converting enzyme inhibitors or angiotensin receptor blockers, as well as traditional medicine. However, despite these efforts, the occurrence and advancement of DKD are not always avoided.

Increasing evidence suggests that vitamin D plays a renal protective role. Vitamin D, a lipid-soluble hormone, binds with specific receptors to help the body maintain calcium and phosphate balance, regulate bone growth, improve cardiovascular function, and protect the kidneys. The mechanisms by which vitamin D protects the kidneys are not fully understood but may involve inhibition of the renin-angiotensin system activity, an increase in the number of podocytes, a reduction in podocyte injury, suppression of pro-inflammatory cytokines (e.g., TGF-β, MCP-1, and TNF-α), and a reduction in oxidative stress levels (21–26). Furthermore, vitamin D levels might improve insulin resistance, promote insulin production, and regulate lipid metabolism, thereby reducing kidney damage (27, 28). Additionally, vitamin D has anti-inflammatory and anti-fibrotic properties, which are crucial in preventing the progression of DKD. Dutta et al. showed that during a two-year follow-up, significantly fewer pre-diabetic patients who supplemented with vitamin D progressed to diabetes mellitus than those who did not supplement, and this result was associated with decreases in inflammatory markers, such as IL-6, TNF-α, and hypersensitive C-reactive protein (29). Moreover, vitamin D can delay the onset of proteinuria. For instance, de Boer et al. analyzed data from the third NHANES survey, finding that as vitamin D levels decreased, the incidence of proteinuria increased, with a relative risk of 1.37 (95% CI, 1.10–1.71) (30). Furthermore, vitamin D’s role in podocyte and tubular autophagy is significant. Autophagy is a cellular process that removes damaged proteins and organelles, thus protecting cells from stress (31). Vitamin D enhances autophagy in renal cells, potentially reducing the risk of DKD.

Globally, vitamin D deficiency is prevalent, with studies indicating that 28–87% of the population in the United States is deficient in vitamin D (32). In Europe, >50% of older adults are vitamin D deficient (33); in the Middle East, severe deficiency rates have been observed to reach up to 60.3% (34). Our study found that 65.1% of the patients with diabetes had insufficient vitamin D levels (<75 nmol/L), and 4.1% had severe deficiency (<25 nmol/L). Our findings, which controlled for various potential risk factors, indicated that serum vitamin D levels were an independent risk factor for DKD in patients with diabetes, particularly in male patients, with higher levels being associated with a reduced risk of DKD; this trend was less pronounced in female patients. Based on these results, a non-linear analysis was conducted, revealing that serum vitamin D levels <59.6 nmol/L increased the risk of DKD, levels between 59.6 and 84.3 nmol/L were protective, and levels >84.3 nmol/L were a risk factor. Our study revealed a U-shaped relationship between serum vitamin D levels and the risk of diabetic kidney disease (DKD) in patients with type 2 diabetes mellitus (T2DM), with optimal vitamin D levels providing a protective effect. Several biological pathways could explain these findings. Vitamin D is known to play a multifaceted role in maintaining kidney health. It helps regulate the renin-angiotensin system (RAS), reducing hypertension, which is a major risk factor for DKD. It is important to note that while vitamin D can be protective within an optimal range, excessive levels of vitamin D may pose a risk for DKD. High levels of vitamin D can lead to hypercalcemia, which can result in calcium deposition in the kidneys, causing nephrocalcinosis and potentially worsening kidney function (35). Moreover, excessive vitamin D can disrupt the balance of calcium and phosphate metabolism, contributing to vascular calcification and increased cardiovascular risk (36). Therefore, maintaining vitamin D levels within an optimal range is crucial to harness its protective benefits while avoiding potential toxicity.

This relationship was more pronounced in male patients, suggesting potential sex-specific mechanisms. Sex-stratified observations indicated a broader protective range for female patients (48–100 nmol/L), compared with male patients (60–80 nmol/L). We examined the relationship between serum vitamin D levels and mortality risk. Our findings revealed that higher serum vitamin D levels are associated with lower mortality risks, particularly among male patients. The sex differences observed in our study could be due to several factors. Estrogen in females enhances vitamin D function by increasing the expression of vitamin D receptors, leading to a more effective anti-inflammatory response (37). Conversely, vitamin D reduces aromatase expression in immune cells, which is responsible for converting testosterone into estrogen, resulting in lower estrogen levels. This difference in hormonal regulation may explain why males benefit more from higher vitamin D levels in terms of reduced DKD risk. Additionally, lifestyle factors and differences in vitamin D metabolism between sexes could contribute to these variations (38).

Our analysis reveals that older age, low family income, and elevated glycated hemoglobin (HbA1c) are significant risk factors for diabetic nephropathy, both in the overall population and across different gender subgroups. Older age is associated with an increased risk of diabetic nephropathy due to the cumulative effects of prolonged hyperglycemia, which can lead to chronic kidney damage over time (39). Aging also results in natural declines in renal function, making the kidneys more susceptible to the detrimental effects of diabetes. Low family income is another critical risk factor, likely due to the associated socioeconomic disadvantages. Individuals with lower income may have limited access to healthcare resources, healthy food options, and diabetes management education, which can lead to poorer glycemic control and, consequently, higher risk of nephropathy (40). Financial constraints can also limit the ability to afford medications and regular medical check-ups, further exacerbating the risk. Elevated HbA1c levels indicate poor long-term glycemic control and are directly linked to the development and progression of diabetic nephropathy. Chronic hyperglycemia can cause damage to the glomeruli in the kidneys, leading to proteinuria and progressive kidney dysfunction. HbA1c is a crucial biomarker for monitoring diabetes management, and persistently high levels reflect the severity of metabolic disturbances that contribute to renal impairment (41). Understanding these risk factors is essential for identifying high-risk individuals and implementing targeted interventions to prevent or delay the onset of diabetic nephropathy. Enhanced screening and tailored management strategies can help mitigate these risks, ultimately improving outcomes for diabetic patients.

As our study is based on NHANES dataset, including a nationally representative sample that is large enough to reflect population disease progression trends, our results provide valuable insights. This comprehensive dataset enabled us not only to investigate the impact of serum vitamin D levels on diabetic kidney disease (DKD) but also to analyze the effects of vitamin D on overall survival among patients with type 2 diabetes mellitus (T2DM). Secondly, we employed restricted cubic spline (RCS) analysis to explore the non-linear relationship between serum vitamin D levels and DKD risk, providing a more nuanced understanding of this complex interaction. Additionally, our study uniquely compared these relationships across different sexes, revealing important sex-specific differences in vitamin D’s protective effects against DKD.

Despite these strengths, there are limitations to our study. As a retrospective analysis, our findings are observational and cannot establish causality. Future research should include longitudinal studies to confirm these associations and further explore the causal mechanisms. One limitation of this study is the potential presence of residual confounders that were not accounted for in the analysis. Although we adjusted for a comprehensive set of covariates, including demographic factors, socioeconomic status, lifestyle behaviors, medical history, and laboratory results, there remain other variables that could influence the relationship between serum vitamin D levels and diabetic kidney disease (DKD). For example, our model did not include dietary intake of vitamin D and calcium, sunlight exposure, physical activity levels, and genetic predispositions (42). These factors are known to affect vitamin D metabolism and could potentially confound the observed relationships. Additionally, the presence of comorbid conditions such as chronic inflammatory diseases or other endocrine disorders, which may alter vitamin D levels and influence DKD risk, were not fully captured (43). Measurement errors in self-reported data, such as dietary intake and physical activity, also exist and could lead to residual confounding. Future studies should aim to include these variables to provide a more accurate assessment of the relationship between vitamin D and DKD.

Furthermore, the use of the NHANES dataset introduces potential measurement and statistical errors that could affect our findings. Measurement errors could arise from inaccuracies in the recorded serum vitamin D levels, which might be influenced by differences in laboratory methods, timing of sample collection, and storage conditions. These inconsistencies can lead to misclassification and bias in the estimated associations. Self-reported data are prone to recall bias and other reporting errors, which can introduce statistical errors and affect the reliability of the findings. Addressing these limitations in future research, perhaps through the use of more standardized measurement protocols and advanced statistical methods, could enhance the robustness and validity of the results.

In conclusion, our study demonstrates the significant role of serum vitamin D levels in influencing the risk of DKD and overall survival in T2DM patients. By utilizing a large, representative database and advanced statistical methods, we provide novel insights into the non-linear and sex-specific effects of vitamin D. Future research should build on these findings to develop more targeted and effective strategies for managing vitamin D levels and preventing DKD in diverse populations.

## Data availability statement

The raw data supporting the conclusions of this article will be made available by the authors, without undue reservation.

## Author contributions

YW: Writing – original draft, Writing – review & editing. CH: Writing – original draft. YL: Investigation, Writing – original draft. QL: Investigation, Writing – original draft. LG: Formal analysis, Writing – original draft. DZ: Formal analysis, Writing – original draft. LC: Formal analysis, Writing – original draft.

## Footnotes

^1^https://www.cdc.gov/nchs/nhanes/index.htm
