# Supplementary material for: Association between serum vitamin D and the risk of diabetic kidney disease in patients with type 2 diabetes
Source: Front Med (Lausanne). 2024 Aug 9;11:1445487. doi: 10.3389/fmed.2024.1445487 (PMC11342449; doi:10.3389/fmed.2024.1445487)
Supplement: Supplementary file 1 [file Data_Sheet_1.docx]

**Supplementary Figure 1. Forest Plot of Multivariate Logistic Regression Analysis Predicting Diabetic Nephropathy Risk in the sensitivity analysis.**


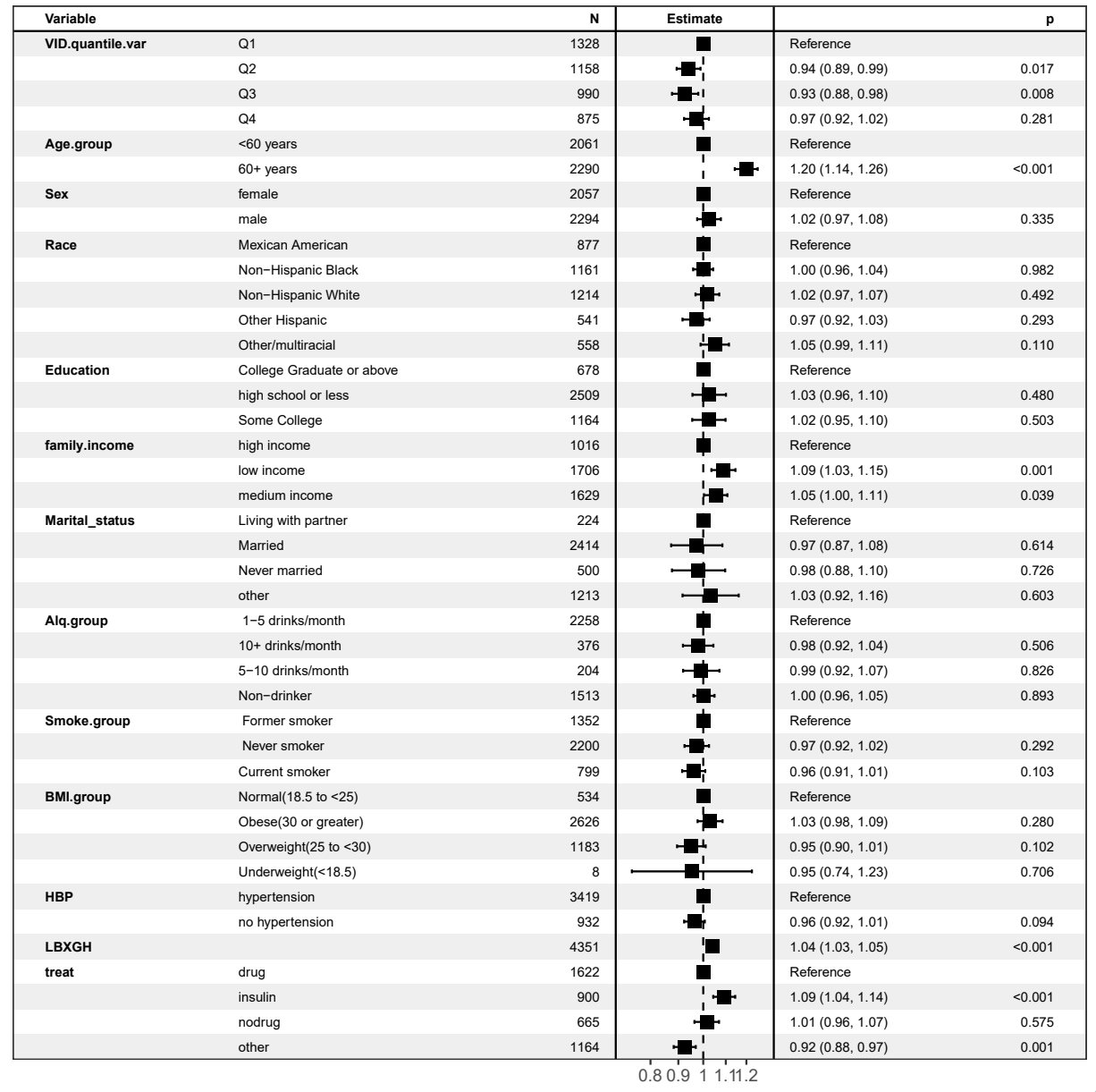


**Supplementary Figure 2. Figure 3: A) Receiver Operating Characteristic Curve for the Entire Population, B) Receiver Operating Characteristic Curve for the Population Under 80 in Sensitivity Analysis.**


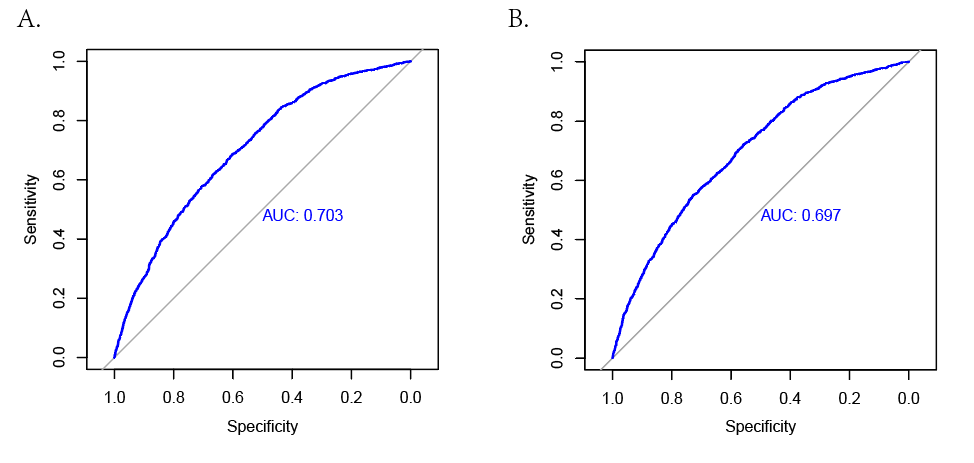


**Supplementary Figure 3: A. Forest Plot of Multivariate Logistic Regression Analysis Predicting Diabetic Nephropathy Risk in Male Group; B: Forest Plot of Multivariate Logistic Regression Analysis Predicting Diabetic Nephropathy Risk in Female Group**

**
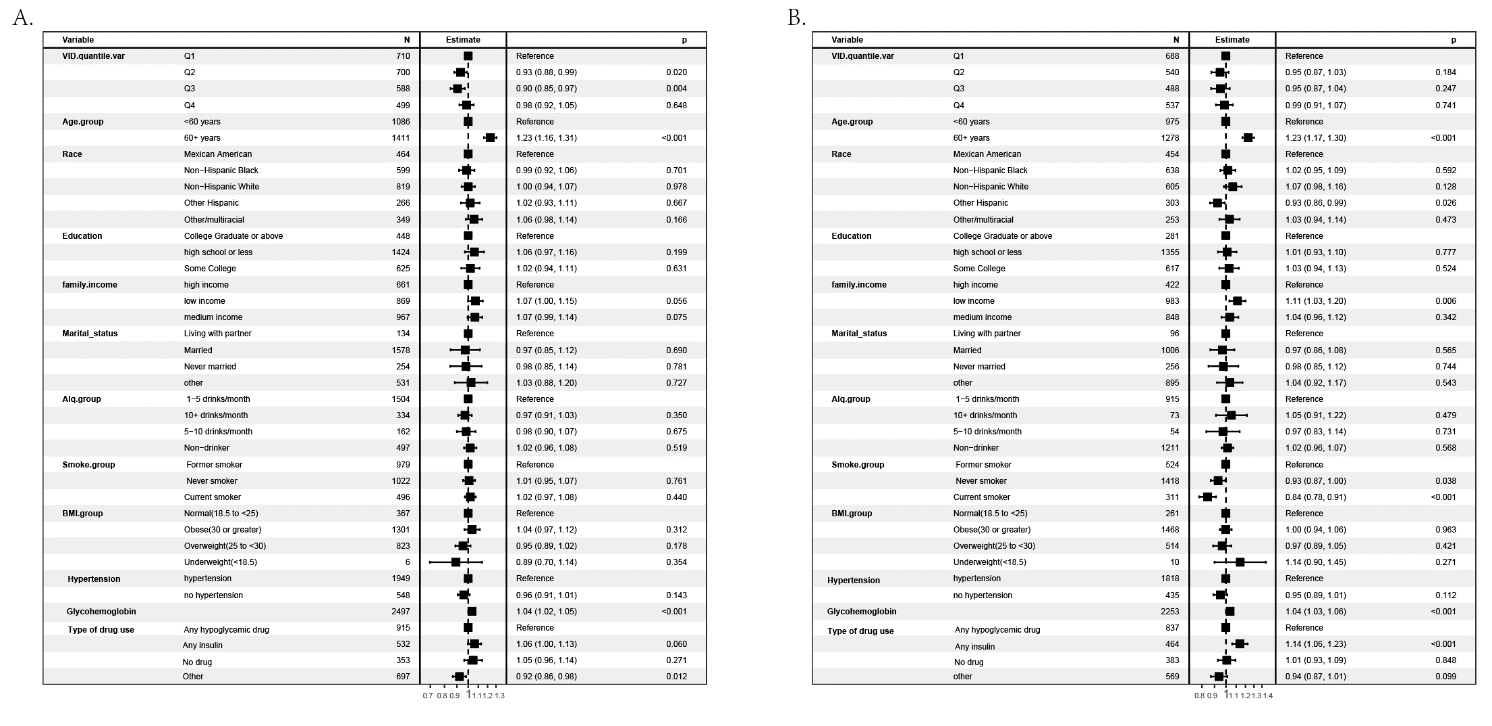
**

**Supplementary figure 4. Correlation between serum vitamin D content and overall survival risk**


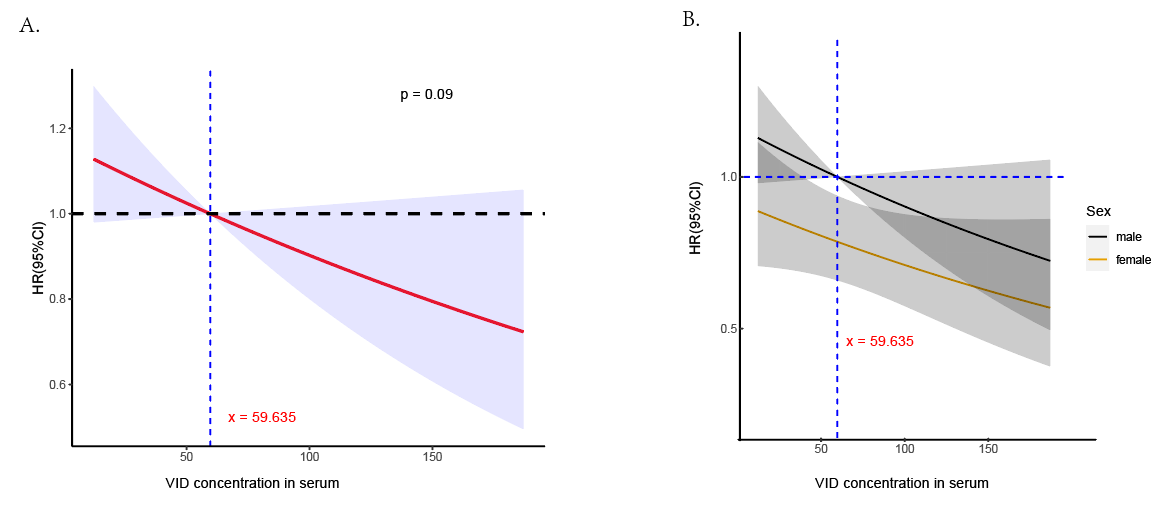


**Supplementary TABLE 1.** The Variance Inflation Factor (VIF) Values for Variables in the Multivariate Logistic Regression of Model2

| Variable | VIF |
| --- | --- |
| VID.quantile.var | 1.34 |
| Age.group | 1.09 |
| Sex | 1.31 |
| Race | 1.33 |

**Supplementary TABLE 2.** The Variance Inflation Factor (VIF) Values for Variables in the Multivariate Logistic Regression of Model3.

| Variable | VIF |
| --- | --- |
| VID.quantile.var | 5.47 |
| Age.group | 1.81 |
| Sex | 3.23 |
| Race | 7.67 |
| Education | 5.81 |
| family.income | 3.09 |
| Marital_status | 5.30 |
| Alq.group | 3.85 |
| Smoke.group | 2.98 |
| BMI.group | 3.53 |
| Hypertension | 1.91 |
| Glycohemoglobin | 1.45 |
| Type of drug use | 4.88 |
